# Exploring the Functional Disorder and Corresponding Key Transcription Factors in Intraductal Papillary Mucinous Neoplasms Progression

**DOI:** 10.1155/2015/197603

**Published:** 2015-09-03

**Authors:** Guiying Bai, Chenxuan Wu, Yingtang Gao, Guiming Shu

**Affiliations:** ^1^Department of Oncology, Tianjin Third Central Hospital, Tianjin 300179, China; ^2^Key Laboratory of Artificial Cell Institute of Hepatobiliary Disease, Tianjin Third Central Hospital, Tianjin 300179, China; ^3^Department of Hepatobiliary Surgery, Tianjin Third Central Hospital, Tianjin 300179, China

## Abstract

This study has analyzed the gene expression patterns of an IPMN microarray dataset including normal pancreatic ductal tissue (NT), intraductal papillary mucinous adenoma (IPMA), intraductal papillary mucinous carcinoma (IPMC), and invasive ductal carcinoma (IDC) samples. And eight clusters of differentially expressed genes (DEGs) with similar expression pattern were detected by *k*-means clustering. Then a survey map of functional disorder in IPMN progression was established by functional enrichment analysis of these clusters. In addition, transcription factors (TFs) enrichment analysis was used to detect the key TFs in each cluster of DEGs, and three TFs (FLI1, ERG, and ESR1) were found to significantly regulate DEGs in cluster 1, and expression of these three TFs was validated by qRT-PCR. All these results indicated that these three TFs might play key roles in the early stages of IPMN progression.

## 1. Introduction 

Intraductal papillary mucinous neoplasms (IPMN) are pancreatic precancerous lesions composed of dilated main and branch ducts and mixed duct type (main and branch ducts are both involved) lined by mucin producing atypical epithelium, which usually proliferates in a papillary fashion [1]. Based on their increasing architectural and nuclear atypia, IPMN are divided into four stages: IPMN low-grade dysplasia (alternatively intraductal papillary mucinous adenoma, IPMA), IPMN intermediate grade dysplasia (alternatively borderline IPMN, IPMB), and IPMN high-grade dysplasia (alternatively intraductal papillary mucinous carcinoma, IPMC) and about 20% to 40% IPMC were invasive ductal carcinoma (IDC) [1, 2]. Moreover, on the basis of the histological and immunohistochemical characteristics, IPMN could be divided into intestinal, pancreatobiliary, oncocytic, and gastric subtypes [3, 4].

The genetic mechanism of IPMN progression has not been completely defined. IPMN are known to harbor mutations of KRAS [5, 6], p16 [6], p53 [5], GNAS [7], RNF43 [8], and DPC4/SMAD4/MADH4 [9], but the prevalence of the mutations is lower than in pancreatic ductal adenocarcinoma (PDAC). With development of high-throughput molecular techniques such as gene microarrays, lots of differentially expressed genes (DEGs) have been found with IPMN progression, such as BRCA1 [10], SOX9 [11], and MUC6 [12]. Meanwhile, some cellular signaling pathways were found to be involved in the IPMN carcinogenesis, such as phosphoinositide 3-kinases-protein kinase B/Akt pathway [13], the DNA damage checkpoint pathway [14], inactivation of tumor suppressor pathways, p53 pathway [15], activation of oncogenic pathways, and K-ras pathway [16]. However, the comprehensive integrated analysis of genes and biological processes and pathways leading to development and progression to malignancy of IPMN were still not clearly demonstrated.

In this study, for comprehensive understanding, the mechanism of IPMN, a microarray dataset including normal main pancreatic ductal tissue (NT), IPMA, IPMC, and IDC originating in IPMN were collected from National Center for Biotechnology Information (NCBI) Gene Expression Omnibus (GEO). Time-series expression analysis was used to screen the multiclass differentially expressed genes (DEGs) during four stages of carcinogenesis. And the DEGs were clustered into different expression pattern clusters using *k*-means clustering. Functional enrichment analysis was used to explore the biological processes and pathways related to each cluster. Then, transcription factors (TFs) enrichment analysis was used to detect the key TFs in each cluster of DEGs. At last, qRT-PCR assay was used to validate the expression of TFs. Finally, an overall survey map of functional disorder and corresponding TFs for the progress of the IPMN were established.

## 2. Materials and Methods

### 2.1. Datasets Used

We selected the gene expression profile data GSE19650 [17] from NCBI GEO, executed on Affymetrix Human Genome U133 Plus 2.0 Array (platform number GPL570); the dataset contains 22 samples including 7 normal main pancreatic ducts (NT), 6 IPMA, 6 IPMC, and 3 IDC originating in IPMN.

Firstly, the raw data were normalized by RMA (robust multiarray average) background subtraction and quantile was normalized using Affymetrix Expression Console Software, and noise derived from absent genes, background, and nonspecific hybridizations was removed. A well-known cancer gene dataset including 547 genes was collected from Cancer Gene Census database [18]. And the significance of overlap between known cancer genes and DEGs was calculated using the cumulative hypergeometric function (*P* < 0.05) (see details in Section 2.4), and calculations were implemented in R statistical package using function phyper (http://www.r-project.org/).

### 2.2. DEGs Screening and Clustering

DEGs among the four stages of IPMN progression were identified by significance analysis of microarrays (SAM) algorithm (multiclass SAM, at least one |fold  change| ≥ 2, *Q*  values < 0.05). Then these DEGs were clustered by *k*-means clustering. Cluster 3.0 software was used to cluster the DEGs clusters and draw cluster picture.

### 2.3. Gene Ontology and Pathway Enrichment Analysis

The functional significance of the DEGs clusters was investigated by performing functional enrichment and clustering analysis using The Database for Annotation, Visualization and Integrated Discovery (DAVID) [19]. One of the DAVID's features is functional annotation cluster in those places similar to GO categories, based on the parent/child GO term associations and the number of the shared genes, into a functional cluster. The GO cluster enrichment score is based on geometric mean of member's *P* values and used to rank their biological significance. So DAVID would report significance of enriched clusters. We used this feature to estimate the relationship between the GO terms (biological process of the GO database) and the KEGG pathways. The threshold of enrichment score for enriched clusters was set as 1 artificially.

### 2.4. TFs Enrichment Analysis

In this study, we downloaded TFs and their regulation genes from TRANSFAC database (Release 3.4) [20]. All experimentally verified TFs and their target gene sets were used in this study, containing 106 TFs, 2921 target genes, and 6238 TF-target gene interactions. Among the 106 TFs, 22 TFs can be mapped to DEGs of IPMN progression. TFs enrichment analysis is a common statistical technique to reveal the significantly changed modules of the target gene sets against the background so as to elucidate the underlying TF regulation mechanisms. Then we enriched DEGs in each cluster with target genes of TFs. The score of each TF and target gene set was calculated using the cumulative hypergeometric function as follows:

(1) $$\begin{array}{c} P=1-\overset{x-1}{\underset{i=0}{\sum }}\frac{\left(\begin{array}{c} K\\ i \end{array}\right)\left(\begin{array}{c} M-K\\ N-i \end{array}\right)}{\left(\begin{array}{c} M\\ N \end{array}\right)}, \end{array}$$

where *M* represents the total number of genes tested. *N* represents the number of genes regulated by a TF. *K* is the number of DEGs in certain cluster. *x* is the number of the DEGs in certain cluster that also appeared in certain TF target genes. The resulting *P* value (*P* < 0.05) reflects the probability of extracting up to *x* of possible *K* genes in *N* drawings. All of the above calculations were implemented in R statistical package using function phyper (http://www.r-project.org/).

### 2.5. qRT-PCR

The tissue samples of four stages of IPMN were collected from Tianjin Third Central Hospital, including five NT, one IPMA, five IPMC, and five IDC originating in IPMN. All IPMA and IPMC samples were classified into gastric type. This study was approved by the Experimental and Ethics Committees of Tianjin Third Central Hospital, and written informed consent from each participating subject had been obtained.

Three TFs (ESR1, ERG, and FLI1) enriched in cluster 1 were validated by quantitative (q) real-time (RT) polymerase chain reaction (PCR) assays using these samples. Primers for the qRT-PCR and annealing temperatures are shown in Table 1. The qRT-PCR reactions were performed on a Roche LightCycler Instrument 1.5, using a LightCycler Fast Start DNA Master PLUS SYBR Green I kit (Roche Cat. 03515885001, Castle Hill, Australia). Briefly, 15 *μ*L reactions: 7.5 *μ*L Master Mix, 0.1 *μ*L forward primer and reverse primer, 1 *μ*L cDNA sample, and 6.3 *μ*L ddH2O were prepared. Each sample was run in triplicate. The RT-PCR program was set to 95°C for 5 minutes and then 45 cycles of 95°C for 10 seconds, 55–60°C for 35 seconds, and 72°C for 40 seconds. At the end of each program, a melting curve analysis was performed. Also, the data were automatically analyzed by the system, and an amplification plot was generated for each cDNA sample at the end of each RT-PCR run. The internal control gene was *β*-actin.

## 3. Results

### 3.1. Identification of DEGs among Four Stages of IPMN Progression

**Figure 1:**
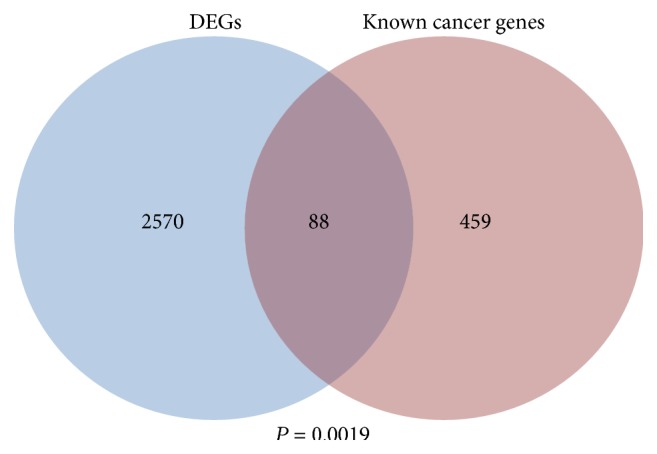
Overlap of DEGs and known cancer genes. The *P* value was calculated by hypergeometric test.

To investigate the gene expression patterns in IPMN progression, multiclass SAM algorithm was used to screen the DEGs among four stages of carcinogenesis, and 2658 DEGs were identified. Then a well-known cancer gene dataset called Cancer Gene Census was used to evaluate the relationship between DEGs and tumorigenesis. Cancer Gene Census is an ongoing effort to catalogue those genes for which mutations have been causally implicated in cancer [18], and many studies used it as a well-known cancer gene dataset [21, 22]. Particularly, there were 88 DEGs (hypergeometric *P* = 0.0019) that overlap with the known cancer genes (see Figure 1), including PDAC related genes such as MYC, BRCA1, and KRAS [23–25], which indicates that the DEGs of this study have close bond with the cancer progression indeed.

### 3.2. Clustering DEGs by Expression Pattern Using *k*-Means Clustering

**Figure 2:**
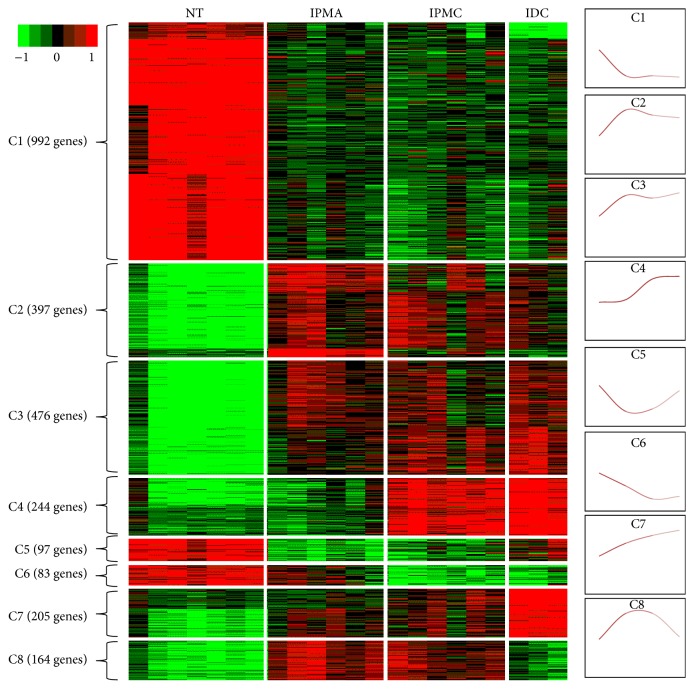
The clustering map of DEGs clusters and expression patterns.

To thoroughly investigate the genomic expression pattern among four stages of IPMN progression, *k*-means clustering was used to cluster the DEGs by expression pattern similarity. As in the results shown in Figure 2, all DEGs were clustering into 8 clusters (named as C1–C8). C1 contained 992 DEGs, which was downregulated in IPMA stage and then kept low expression level in IPMC and IDC stages; C2 contained 397 DEGs, which was upregulated in IPMA stage and then slightly downregulated during IPMC and IDC stages; C3 contained 476 DEGs, which was upregulated in IPMA stage, slightly downregulated in IPMC stage, and then slightly upregulated in IDC stage; C4 contained 244 DEGs, which kept low expression level in IPMA stage and was upregulated in IPMC stage and then slightly upregulated in IDC stage; C5 contained 97 DEGs, which was downregulated in IPMA stage, slightly upregulated in IPMC stage, and then upregulated in IDC stage; C6 contained 83 DEGs, which was downregulated in IPMA and IPMC stages and then kept low expression level in IDC stage; C7 contained 205 DEGs, which was continuously upregulated during the three stages of IPMN; C8 contained 164 DEGs, which was upregulated in IPMA stage and kept high expression level in IPMC stage and was then downregulated in IDC stage. For all these clusters of DEGs, different clusters were regulated in different cancer stages, which indicates that each cluster may play roles in one or several specific cancer stages.

### 3.3. Functional Enrichment Analysis of 8 DEGs Clusters

**Figure 3:**
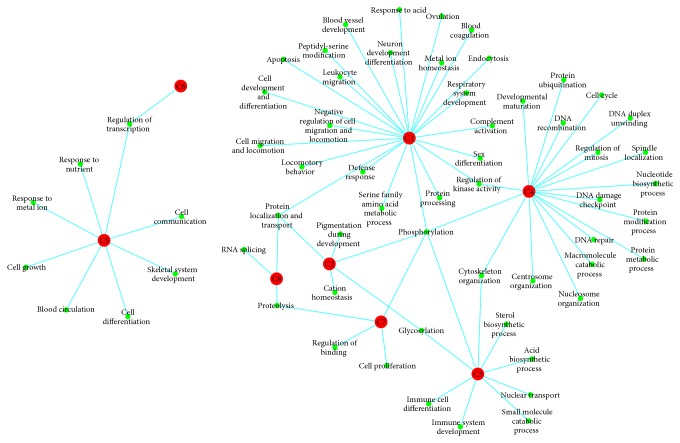
The biological processes and pathways related to DEGs clusters. Red node: cluster of DEGs; green node: biological process; line: relationship between cluster and biological process.

Analysis of DEGs clusters could help pinpoint potential functional mechanism important for gene expression during IPMN progression [26]. Therefore, DAVID was used to evaluate the biological processes and pathways related to these 8 clusters of DEGs (enrichment score > 1). As shown in Figure 3 and Supplementary Tables in Supplementary Material available online at http://dx.doi.org/10.1155/2015/197603, DEGs in C1 that contains almost half of all DEGs were enriched in many biological processes, mainly involved in 5 aspects: “vasculature development,” “cell migration and locomotion,” “neuron development differentiation,” “phosphorylation,” and “endocytosis”; DEGs in C2 were enriched in “phosphorylation,” “pigmentation during development,” “cation homeostasis,” “protein localization and transport,” and “glycosylation”; DEGs in C3 were enriched in “acid biosynthetic process,” “immune cell differentiation,” “cytoskeleton organization,” “glycosylation,” “small molecule catabolic process,” and “nuclear transport”; DEGs in C4 were enriched in “cell cycle,” “mitosis,” “DNA and chromosome organization,” “protein ubiquitination,” and “phosphorylation”; DEGs in C5 were enriched in “cell growth, development, and differentiation,” “skeletal system development,” “cell communication,” “blood circulation,” and “response to nutrient, metal ion”; DEGs in C6 were enriched in “regulation of transcription”; DEGs in C7 were enriched in “phosphorylation,” “proteolysis,” “cell proliferation,” and “regulation of binding”; DEGs in C8 were enriched in “RNA splicing,” “protein localization and transport,” and “proteolysis.” Among these various biological processes, most of them were involved in multiple cancer stages such as “phosphorylation,” “glycosylation,” and “proteolysis”; for comparison, some biological processes played a role in specific cancer stage; for example, “cell cycle” and related functions play a main role in C4, in other words, between IPMA and IPMC stage.

### 3.4. TFs Enrichment Analysis of 8 Clusters of DEGs

**Figure 4:**
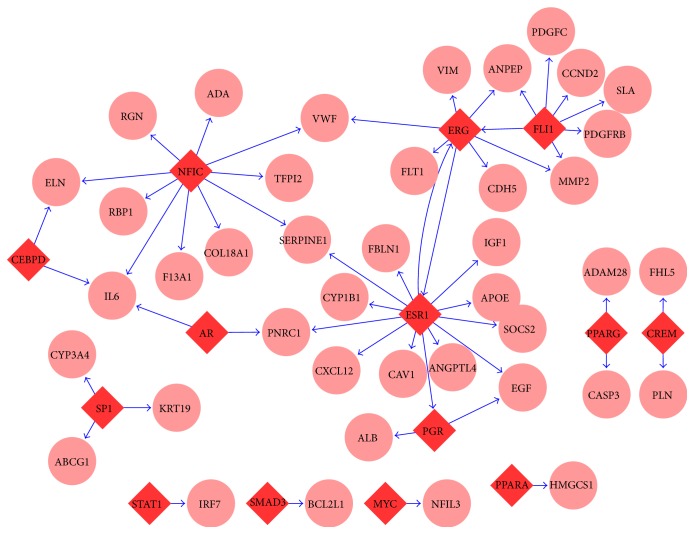
The TF and target genes networks in different clusters of DEGs. Red diamond: TF; salmon circle: target gene; blue line: regulatory relationships.

The cluster analysis of DEGs identified groups of genes that exhibit similar expression profiles and thus might be coregulated. Analysis of such coregulated gene sets could help pinpoint potential TFs important for gene expression during cancer progression [27]. Therefore, to gain an initial understanding of the regulatory mechanisms of genes differentially expressed during cancer progression, a total of experimentally validated TFs network data containing 106 TFs, 2921 target genes, and 6238 TF-target gene interactions were gathered from TRANFAC database [20]. Genes in each cluster were screened for overrepresentation of known TFs (cumulative hypergeometric; see Section 2 for details). 14 TFs which have target genes in these clusters were detected, and TFs enrichment analysis showed that three TFs (ESR1, ERG, and FLI1) were selectively enriched in cluster 1 (as shown in Table 2 and Figure 4), suggesting that these three TFs and their corresponding target genes may play important roles in early stages of IPMN progression, but these three TFs in IPMN progression have not yet received much attention and research. In addition, the rest of 11 TFs such as SAMD4, MYC, PPARG, and PPARA have also been proved to play key roles in various cancer progressions [28–30].

### 3.5. Expression of Three TFs

**Figure 5:**
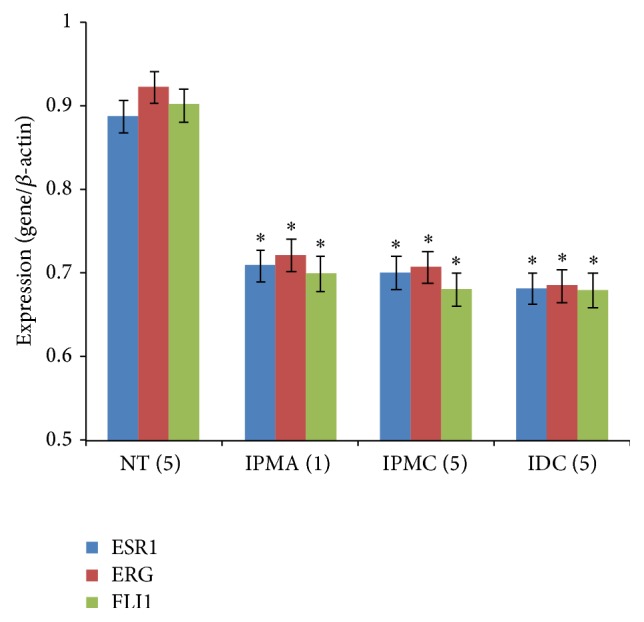
Expression of three TFs in different stages of IPMN samples. ^*∗*^ *P* < 0.05 as determined by a Student's *t*-test compared with the NT samples.

The qRT-PCR experiments were used to verify the expression of these three TFs in different stages of IPMN. As in the results showed in Figure 5, the mRNA levels of ESR1, ERG, and FLI1 were remarkably decreased between NT and IPMA (*t*-test, *P* < 0.05) and then continuously and slightly decreased during IPMN progression, which confirmed the results of the microarray analysis and TFs enrichment analysis. These results suggested that these three TFs may play important roles at the beginning of IPMN and still have some effect in the process of IPMN progress to IDC.

## 4. Discussion 

Intraductal papillary mucinous neoplasm (IPMN) is a kind of PDAC, about 40% of which may develop into IDC [31], and the 5-year survival rate for patients with these lesions varied widely from 0% to 64% in several reported series [32–34]. The progression could be divided into four stages, ranging from adenoma to invasive carcinoma by neoplasm dysplastic changes. Therefore, revelation of the molecular mechanism of the multistep carcinogenesis process carried out for clinical treatment and diagnosis has an important role in guiding. In this study, for comprehensive investigating of the IPMN progression, a time-series clustering analysis method was performed to analyze the genomic expression patterns of multistages of IPMN; then functional and TFs enrichment analysis were used to identify the highly related function and molecular mechanism of IPMN progression. The pipeline of these bioinformatics methods might suggest lessons for other studies.

**Figure 6:**
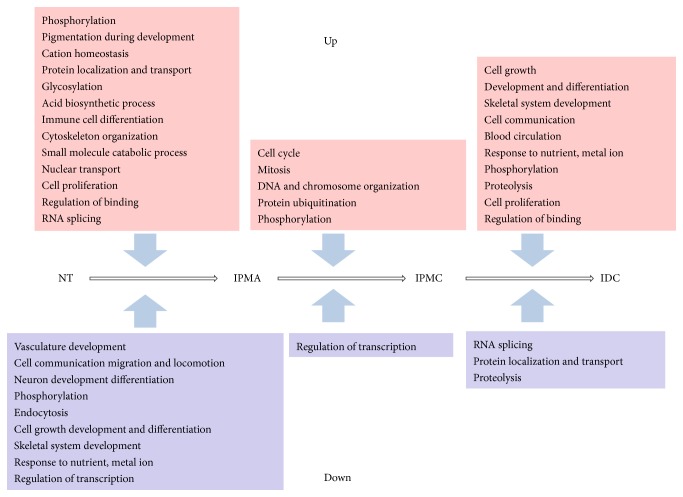
The overall survey map of functional disorder for IPMN progression.

From the results of clustering and functional enrichment analysis of DEGs, eight clusters of DEGs with different expression patterns and the corresponding various biological processes and pathways were identified. And an overall survey map of functional disorder for the progression has been established according to these results. As Figure 6 showed, from NT to IPMA stage, many upregulated DEGs (DEGs in C2, C3, C7, and C8) were enriched in various biological processes such as “phosphorylation,” “cell proliferation,” and “cytoskeleton organization,” and lots of downregulated DEGs (DEGs in C1, C5, and C6) were enriched in biological processes such as “cell communication, migration, locomotion, growth, development, and differentiation” and “vasculature development.” These biological processes have been proved to have significant relationships with pancreatic cancer progress [35–37]. In accord with the clinical characteristics of IPMA, these results indicate that, in the early stage (IPMA) of carcinogenesis, the pancreatic duct cell began proliferation, development, and differentiation and obtained the migration ability by communicating with other cells, transferring the signaling by protein phosphorylation activity. These molecule changes may lead to dilatation and/or cystic lesion of the pancreatic duct and show low-grade dysplasia. Meanwhile, for supply nutrients to development of dilatation and/or cyst, these processes were accompanied with vasculature development. From IPMA to IPMC stage, the up- (DEGs in C4) and downregulated DEGs (DEGs in C6) were enriched in biological processes such as “mitosis, cell cycle,” “phosphorylation,” and “protein ubiquitination,” suggesting that the adenoma cell suffers severe cell cycle disorder, accompanied with drastic protein phosphorylation and ubiquitination. These molecule changes may make cells of dilatation and/or cyst grow up rapidly and show intermediate grade and high-grade dysplasia. From IPMC to IDC, the up- (DEGs in C5 and C7) and downregulated DEGs (DEGs in C8) were enriched in biological processes such as “cell growth, proliferation, and communication,” “RNA splicing,” “protein localization and transport,” and “proteolysis.” These processes indicate that, in IDC stage, the cancer cell obtained ability of cell growth and proliferation. Moreover, dilatation and/or cyst may obtain invasiveness and become malignant by protein phosphorylation and cell-cell communication. In this stage, the gene transcription and protein metabolism increased, invasive cells proliferated enormously, and IPMC developed to IDC. For this survey map, a biological process, phosphorylation, has been found to be involved in all stages, which indicates that this process may play a key role in the whole progression of carcinogenesis and may be a new targeted therapeutic strategy for IPMN.

The cluster analysis of DEGs identified groups of genes that exhibit similar expression profiles and thus may be coregulated. Analysis of such coregulated gene sets could help pinpoint potential TFs important for gene expression during cancer progression. Therefore, TFs and their corresponding target genes dataset was gathered from TRANSFAC database for enrichment analysis. Three TFs (ESR1, ERG, and FLI1) were detected that not only significantly regulate the DEGs in cluster 1, but also have regulatory relationships between each other. What is more, the results of qRT-PCR experiments validated that these three TFs were remarkably decreased between NT and IPMA and then continuously and slightly decreased during IPMN progression. ERG and FLI1 genes are closely related members of the erythroblast transformation-specific (ETS) family of transcription factors, and studies have proved that these two TFs were usually involved in various types of cancer jointly [38, 39]. Meanwhile, the ESR1, estrogen receptor encoding gene, has been proved to be involved in pathological processes including breast cancer and endometrial cancer [40]. But until now no studies have focused on these 3 TFs in IPMN. Moreover, most of the regulated genes (18/25) of these three TFs could be found in Pancreatic Cancer Database [41]. Nevertheless, our study indicates that these three TFs may be the key regulators in early stage of IPMN and are definitely worth further exploration. In addition, the rest of TFs such as PPARG, PPARA, SMAD3, MYC, and STAT1 have been proved to be related to cancer progression [28–30, 42].

## 5. Conclusion

Our study provides a comprehensive perspective for the progression of pancreatic carcinogenesis by distinct bioinformatics analysis. A number of DEGs clusters and specific biological processes were detected in progression, and then an overall survey map of functional disturbance for the progression has been established. Then, the TFs enrichment analysis and qRT-PCR experiments found that three TFs may be playing key roles in early stages of IPMN progression. We believe that these results have a certain value in development of IPMN mechanism study and based medicine research.

## Supplementary Material

Supplementary Tables. functional enrichment analysis for 8 clusters of DEGs. DAVID was used to evaluate the biological processes and pathways related to these 8 clusters of DEGs. Meanwhile, we estimated the relationship between the GO terms (biological process of the GO database) and the KEGG pathways. The threshold of enrichment score for enriched clusters was set as 1.

## Figures and Tables

**Table 1:** The PCR primers of gene.

Gene	Sequence (5′ → 3′)	Length
*β*-actin	Forward primer	CATGTACGTTGCTATCCAGGC
	Reverse primer	CTCCTTAATGTCACGCACGAT

ESR1	Forward primer	GGGAAGTATGGCTATGGAATCTG
	Reverse primer	TGGCTGGACACATATAGTCGTT

ERG	Forward primer	CGTGCCAGCAGATCCTACG
	Reverse primer	GGTGAGCCTCTGGAAGTCG

FLI1	Forward primer	CCAACGAGAGGAGAGTCATCG
	Reverse primer	TTCCGTGTTGTAGAGGGTGGT

**Table 2:** The TFs and target genes in different clusters.

Cluster	TF	Target genes	*P* value
C1	FLI1	ANPEP, PDGFRB, SLA, ERG, PDGFC, MMP2, CCND2	**0.0041**
	ERG	ANPEP, CDH5, ESR1, FLT1, MMP2, VIM, VWF	**0.0372**
	ESR1	CXCL12, IGF1, ANGPTL4, APOE, ERG, SOCS2, CYP1B1, CAV1, EGF, PNRC1, PGR, FBLN1, SERPINE1	**0.0451**
	NFIC	VWF, COL18A1, ELN, IL6, TFPI2, ADA, RGN, SERPINE1, RBP1, F13A1	0.1671
	PGR	ALB, EGF	0.3925
	CEBPD	ELN, IL6	0.5027
	AR	IL6, PNRC1	0.4326
	CREM	FHL5, PLN	0.5806

C3	PPARG	CASP3, ADAM28	0.3851
	PPARA	HMGCS1	0.7016

C5	MYC	NFIL3	0.8412

C7	SP1	ABCG1, CYP3A4, KRT19	0.2409
	STAT1	IRF7	0.6291
	SMAD3	BCL2L1	0.4531
